# VNLG-152R and its deuterated analogs potently inhibit/repress triple/quadruple negative breast cancer of diverse racial origins *in vitro* and *in vivo* by upregulating E3 Ligase Synoviolin 1 (SYVN1) and inducing proteasomal degradation of MNK1/2

**DOI:** 10.3389/fonc.2023.1240996

**Published:** 2023-09-11

**Authors:** Retheesh S. Thankan, Elizabeth Thomas, Puranik Purushottamachar, David J. Weber, Vidya P. Ramamurthy, Weiliang Huang, Maureen A. Kane, Vincent C. O. Njar

**Affiliations:** ^1^ Department of Pharmacology, University of Maryland School of Medicine, Baltimore, MD, United States; ^2^ The Center for Biomolecular Therapeutics, University of Maryland School of Medicine, Baltimore, MD, United States; ^3^ Isoprene Pharmaceuticals, Inc., Baltimore, MD, United States; ^4^ Marlene and Stewart Greenebaum Comprehensive Cancer Center, University of Maryland School of Medicine, Baltimore, MD, United States; ^5^ Department of Biochemistry and Molecular Biology, University of Maryland School of Medicine, Baltimore, MD, United States; ^6^ Department of Pharmaceutical Sciences, University of Maryland School of Pharmacy, Baltimore, MD, United States

**Keywords:** triple/quadruple negative breast cancer (TNBC/QNBC), MNK1 and MNK2 degrader, eIF4E signaling, Synoviolin 1 (SYVN1), VNLG-152R and deuterated analogs

## Abstract

Triple-negative breast cancer (TNBC) and its recently identified subtype, quadruple negative breast cancer (QNBC), collectively account for approximately 13% of reported breast cancer cases in the United States. These aggressive forms of breast cancer are associated with poor prognoses, limited treatment options, and lower overall survival rates. In previous studies, our research demonstrated that VNLG-152R exhibits inhibitory effects on TNBC cells both *in vitro* and *in vivo* and the deuterated analogs were more potent inhibitors of TNBC cells *in vitro.* Building upon these findings, our current study delves into the molecular mechanisms underlying this inhibitory action. Through transcriptome and proteome analyses, we discovered that VNLG-152R upregulates the expression of E3 ligase Synoviolin 1 (SYVN1), also called 3-hydroxy-3-methylglutaryl reductase degradation (HRD1) in TNBC cells. Moreover, we provide genetic and pharmacological evidence to demonstrate that SYVN1 mediates the ubiquitination and subsequent proteasomal degradation of MNK1/2, the only known kinases responsible for phosphorylating eIF4E. Phosphorylation of eIF4E being a rate-limiting step in the formation of the eIF4F translation initiation complex, the degradation of MNK1/2 by VNLG-152R and its analogs impedes dysregulated translation in TNBC cells, resulting in the inhibition of tumor growth. Importantly, our findings were validated *in vivo* using TNBC xenograft models derived from MDA-MB-231, MDA-MB-468, and MDA-MB-453 cell lines, representing different racial origins and genetic backgrounds. These xenograft models, which encompass TNBCs with varying androgen receptor (AR) expression levels, were effectively inhibited by oral administration of VNLG-152R and its deuterated analogs in NRG mice. Importantly, in direct comparison, our compounds are more effective than enzalutamide and docetaxel in achieving tumor growth inhibition/repression in the AR+ MDA-MD-453 xenograft model in mice. Collectively, our study sheds light on the involvement of SYVN1 E3 ligase in the VNLG-152R-induced degradation of MNK1/2 and the therapeutic potential of VNLG-152R and its more potent deuterated analogs as promising agents for the treatment of TNBC across diverse patient populations.

## 1 Introduction

Breast cancer is a significant global health concern and a leading cause of cancer-related mortality among women worldwide. It is the second main cause of cancer-related death in the American women and the most detected cancer in women globally (1). Over the past three decades, the rate of incidence has been increasing by 0.3% every year though the death rate decreased significantly due to advanced medical intervention (1). Among all the subtypes, triple negative breast cancer (TNBC) and lately classified quadruple negative breast cancer (QNBC) are highly resilient and elude currently available treatment strategies (2). While TNBC lacks the expression of estrogen receptor (ER), progesterone receptor (PR) and expresses low levels of HER2, QNBC is characterized by low or no androgen receptor (AR) expression apart from the features of TNBC (3). More than 57% of TNBC diagnosed lack AR expression and may be sub-categorized as QNBC (2, 4). As TNBC and QNBC lack important pharmacological targets, both these subtypes are therapeutically challenging and highly metastatic in nature (5). Therefore, the development of novel therapeutic drugs that effectively inhibits TNBC/QNBC is an urgent ongoing medical need (6).

Interestingly, the TNBC subtype Luminal AR (LAR) that expresses AR is significantly driven by AR signaling and associated with decreased disease-free survival and poor overall survival (7). Meta-analyses of AR expression in TNBC reveals that 27.96% of the 4703 patients studied expressed AR (8). In the clinical trial to identify AR-positive TNBC patients, 80% of the 368 patients screened expressed AR and responded to AR inhibitor enzalutamide (7). Therefore, AR is considered a significant pharmacological target in combating TNBC. However, in the absence of AR in other sub-types such as QNBC, targeting other pathways such as MNK-eIF4E and mTORC1 is more rational (9). Phosphorylation of eIF4E by MNK1/2 is a critical step in mRNA 5’cap-dependent translation of many proteins that actively promote cell division and tumor growth (10).

Pharmacological targeting of oncogenic eIF4F translation initiation complex has been an attractive therapeutic strategy for the development of novel drugs to treat various cancers (11, 12). EIF4E, being the least abundant protein of the eIF4F complex is considered the rate-limiting factor in mRNA 5’-cap-dependent translation initiation (13). Phosphorylation of eIF4E is critical for the formation of eIF4F. MNK1 and MNK2 are the only kinases known to phosphorylate eIF4E when both are bound to the scaffolding protein eIF4G to form the translation initiation complex eIF4F (13–15). Further, MNK1/2 being at the center of eIF4E signaling and mTORC signaling (16), pharmacological inhibition of MNK1/2 is a potent strategy to combat various cancers including TNBC (17).

**Figure 1:**
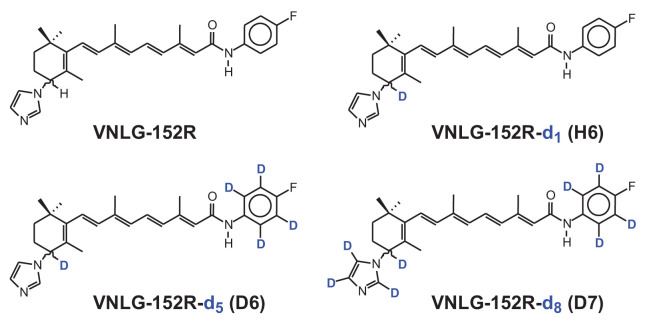
The chemical structures of VNLG-152R and its deuterated analogs D6, D7 and H6. The hydrogen atoms in the indicated positions were replaced by the heavy isotope deuterium to improve the pharmacokinetic properties and retention time in plasma for enhanced antitumor efficacy.

Previously, we reported the development of a novel MNK1/2 degrader VNLG-152R that promotes degradation of MNK1/2 in breast cancer cells (18) and inhibits TNBC *in vivo* (9). Additionally, we explored the potential of deuterated derivatives of VNLG-152R, which showed enhanced efficacy against TNBC cells *in vitro* and improved pharmacokinetic properties in mice models (19). The incorporation of deuterium, by replacing hydrogen atoms, has emerged as a promising strategy to enhance pharmacokinetic and therapeutic profiles of various drugs (20). The deuterated analogs were either better or equipotent to VNLG-152R in *in vitro* antiproliferative activities against MDA-MB-231 and MDA-MB-468 human TNBC cells. Importantly and as expected, the expression of Mnk1, peIF4E and their associated downstream targets, including cyclin D1 and Bcl2, were strongly decreased in VNLG-152R/deuterated analogs-treated TNBC cells signifying inhibition of Mnk1-eIF4E signaling (*i.e.*, target engagement). Among the seven deuterated analogs of VNLG-152R examined, three novel analogs (D6, D7 and H6) (**Figure 1**) exhibited enhanced pharmacokinetic parameters including prolonged residence time and extended elimination half-life in plasma in CD-1 female mice (19). These findings highlight the potential of deuterated analogs as promising candidates for further development in the treatment of TNBC.

In this study, we unveil the key molecular mechanism behind degradation of MNK1/2 by VNLG-152R in breast cancer cells. Our findings highlight the upregulation of Synoviolin 1 (SYVN1), also called 3-hydroxy-3-methylglutaryl reductase degradation (HRD1), an E3 ligase by VNLG-152R and its significant role in ubiquitination and proteasomal degradation of MNK1/2. To broaden our investigation, we conducted a comprehensive evaluation comparing the *in vivo* efficacy of VNLG-152R’s deuterated analogs, namely D6, D7 and H6, with the parent compound in three different tumor xenografts of MDA-MB-231 (derived from Caucasian female metastatic mammary adenocarcinoma, low AR/AR^‐^), MDA-MB-468 (derived from metastatic mammary adenocarcinoma of an African female patient and AR^‐^), and MDA-MB-453 of Caucasian female origin with high AR expression. Additionally, we compared the efficacies of VNLG-152R and the most potent deuterated analog, D7, to the efficacies of clinically relevant TNBC drugs, such as docetaxel (DTX) and enzalutamide (ENZ), in mice tumor xenografts MDA-MB-453 of Caucasian female origin with high AR expression (21, 22).

We must acknowledge the emerging racial disparities in TNBC occurrence and subsequent mortality rates, with women of African descent facing higher vulnerability (23). Hence, our investigation also encompassed the evaluation of VNLG-152R and its deuterated analogs in three distinct tumor xenografts representing diverse racial origins, including both Caucasian and African women, while considering their respective AR expression status. Through our comprehensive study, we aim to shed light on the intricate molecular mechanisms underlying TNBC and address the urgent need for effective therapeutic interventions tailored to specific racial populations. These findings hold promise in advancing personalized medicine approaches for the treatment of TNBC, ultimately contributing to the overall improvement of patient outcomes irrespective of their ethnicity.

In the current study, we employed *in vitro*, *in vivo*, molecular, and biochemical approaches to investigate the effects of VNLG-152R and its deuterated analogs on TNBC. Next-generation RNA-sequencing, differential gene expression analysis and HD Mass Spectrometry Proteome revealed significant upregulation of SYVN1, in response to VNLG-152R treatment. Furthermore, the modulation of multiple pathways by VNLG-152R contributed to the inhibition of TNBC. Biochemical analyses confirmed the presence of elevated levels of SYVN1 protein in both VNLG-152R-treated cells *in vitro* and tumor tissues from the treated mice. Notably, we show for the first time that VNLG-152R facilitated the ubiquitination of MNK1/2 by SYVN1, leading to its subsequent proteasomal degradation, which ultimately contributed to the inhibition of TNBC. Degradation of MNK1/2 further affected phosphorylation of eIF4E adversely, which in turn restricted mRNA 5’cap-mediated translation initiation thereby checking uncontrolled protein synthesis in tumor cells, effectively restricting tumor growth and proliferation.

## 2 Materials and methods

### 2.1 Cell culture, western blotting, and fine chemicals

The human breast cancer cell lines, MDA-MB-231, MDA-MB-468 and MDA-MB-453 representing triple negative breast cancer of Caucasian origin with no AR expression (QNBC), African origin with low AR and Caucasian origin with high AR expression respectively obtained from ATCC (Manassas, VA) were cultured in the recommended media supplemented with 10% heat-inactivated standard fetal bovine serum (FBS, GIBCO) and 1% penicillin-streptomycin (10,000 U/ml, Life Technologies) at 37°C and 5% CO_2_. Primary antibodies of MNK1, MNK2, eIF4E, p-eIF4E, SYVN1, Ubiquitin, β-actin, GAPDH and secondary HRP-conjugated anti-rabbit were obtained from Cell Signaling Technology, USA. The cells were lysed in radioimmunoprecipitation assay (RIPA) buffer supplemented with 1x protease inhibitors (Roche, Indianapolis, IN, USA), phosphatase inhibitors (Thermo Scientific, Waltham, MA, USA), 1 mmol/L EDTA and 1 mmol/L PMSF (Sigma) and immunoblotted as described earlier (24, 25). Immunoprecipitation of MNK1 was performed as reported previously using MNK1 primary antibody (26, 27). All fine chemicals were purchased from Sigma-Aldrich, St. Louis, MO. VNLG-152R and the deuterated analogs (D6, D7 and H6) were synthesized in house as described previously (19). The chemical structures of VNLG-152R and its deuterated analogs are presented in **Figure 1**.

### 2.2 RNA-sequencing and GSEA

MDA-MB-231 cells were treated with 10 μM VNLG-152R for 24 h in triplicates. Total RNA was isolated using RNAeasy Plus mini kit (Qiagen) following manufacturer’s instructions. The RNA preparation was quantified and assessed its quality using Agilent 2100 Bioanalyzer. A RIN value of 8 or above was used for all samples. The sequencing libraries were prepared with the NEB Ultra II Directional RNA library prep kit. Further, the libraries were evaluated for quantity and size distribution using Qubit and Agilent 2100 Bioanalyzer. Sequencing was carried out on an Illumina NovaSeq S2 PE100 bp lane (Maryland Genomics, Institute for Genome Sciences, University of Maryland Baltimore). As a norm, Phred quality score (Q score; to measure the quality of sequencing) more than 90% of the sequencing reads reached Q30 (99.9% base call accuracy). Differential Gene Expression and Gene Set Enrichment analyses (GSEA) were performed to identify canonical cellular pathways modulated by VNLG-152R as reported previously (28).

### 2.3 siRNA-mediated knockdown of gene expression

Specific siRNA targeting SYVN1 and scramble siRNA (siControl) were purchased from Ambion (Foster City, CA, USA). MDA-MB-231 and MDA-MB-468 cells were grown in 6-well culture plates and transfected with siRNA using lipofectamine RNAiMax transfection reagent (Invitrogen, USA) in Opti-MEM reduced serum medium (Thermo Fisher Scientific, USA) for 48 h according to the manufacturer’s protocol. Scrambled siRNA was transfected as control and SYVN1 knockdown was scored by immunoblot analyses.

### 2.4 Proteome profiling by high-definition mass spectrometry

MDA-MB-231 cells treated with VNLG-152R (10 μM, 24 h) or vehicle control were lysed in 4% deoxycholate and the lysates were washed, reduced and alkylated followed by trypsin-lysis as described (29). The tryptic fragment peptides were separated in a nanoACQUITY UPLC analytical column (BEH130 C18, 1.7 μm, 75 μm x 200 mm, Waters) over a 180 min linear acetonitrile gradient (3–43%) containing 0.1% formic acid in nano-ACQUITY UPLC system, Waters Corporation and analyzed in coupled Waters Synapt G2S HDMS mass spectrometry system. The spectra acquired using ion mobility linked parallel mass spectrometry (UDMSe) were analyzed as reported previously (30, 31).

Tandem mass spectra generated were aligned using UniProt human reference proteome. The resulting hits were further validated at a maximum false discovery rate of 0.01. The abundance ratio between the control and VNLG-152R treatments were calculated by comparing the MS1 peak volumes of peptide ions at the low collision energy cycle. The MS1 peptides were further validated by MS2 sequencing at higher collision energy cycle. Label-free quantifications were performed using aligned AMRT (Accurate Mass and Retention Time) cluster quantification as reported previously (32).

### 2.5 *In vivo* tumor xenograft studies

All animal studies in mice were performed in accordance with the humane use of experimental animals following review and approval by the Institutional Animal Care and Use Committee (IACUC), University of Maryland School of Medicine, Baltimore, MD, USA, per IACUC No. # 0221010 dated 03/09/2021. The human breast cancer cell lines, MDA-MB-231, MDA-MB-468 and MDA-MB-453 representing triple negative breast cancer of diverse ethnic origin and AR expression status were used to induce tumor xenografts in immunodeficient female NRG mice (age 5–7 weeks) procured from the Veterinary Resources, University of Maryland School of Medicine, Baltimore, MD, USA. The animals were housed under sterile conditions and fed with sterile pellets and water *ad libitum*. After a week of acclimatization, 3-5x10^6^ cells in 100 μl were subcutaneously injected into the left flank of mice. After 21-25 days of inoculation and upon reaching the tumor volume ~100 mm^3^, the animals were randomly grouped into five animals per group. The control animals were orally administered with vehicle (20% β-cyclodextrin in saline, PO) and other compounds administered (PO or IP as indicated) with indicated doses of test compounds and duration. The animals were carefully observed daily for general health and body weight recorded three times a week. The tumor size was measured three times a week using digital calipers and tumor volume calculated using the formula length (mm) x width^2^ (mm) x 0.5 (mm^3^). Upon reaching a tumor length of approximately 20 mm or a tumor volume of 2000 mm^3^, whichever was achieved first in the control groups (approximately 6 weeks after breast cancer cell inoculation), the study was promptly concluded. Subsequently, the mice were humanely euthanized, and the tumors were surgically removed for further analysis.

### 2.6 Statistical analysis

Statistical comparisons were made by one-way ANOVA followed by Multiple comparisons test using GraphPad Prism 9.0 software (GraphPad Software, Inc.). A probability value with *p < 0.05, **p<0.001 and ***p<0.0001 were considered statistically significant. As specified in the figures, values in data are expressed as the mean ± SEM of three or more independent experiments.

## 3 Results

### 3.1 MNK1/2 and eIF4E are upregulated in breast cancer: TCGA and CPTAC database

**Figure 2:**
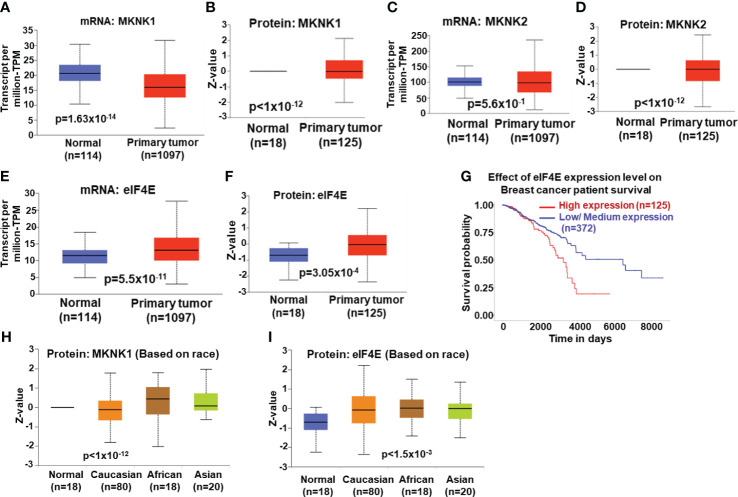
Expression (mRNA) and protein levels of MNK1/2 and eIF4E are significantly high in tumor tissues of breast cancer patients: TCGA (The Cancer Genome Atlas) and CPTAC (Clinical Proteomic Tumor Analysis Consortium). **(A-F)** The mRNA levels in tumor tissues of 1097 breast cancer patients were compared to the mRNA levels in the adjacent normal tissues of 114 individuals. The protein levels of MNK1/2 and eIF4E were analyzed from tumor tissues from 125 patients against that of normal tissue from 18 individuals. Both mRNA and protein levels of MNK1/2 and eIF4E are significantly higher in tumor tissue from breast cancer patients compared to the adjacent normal tissue. **(G)** Increased level of eIF4E is correlated to poor overall survival rate in breast cancer patients. **(H, I)** Protein level of MNK1 and eIF4E is significantly high in clinical tumor specimens of African and Caucasian races respectively and are likely to benefit from therapies targeting MNK1/2-eIF4E signaling.

Notably, transcriptome and proteome analyses using data from The Cancer Genome Atlas (TCGA) and Clinical Proteomic Tumor Analysis Consortium (CPTAC) have revealed consistent upregulation of MNK1/2 and eIF4E in most breast cancer cases at mRNA and protein level except MNK1 mRNA (**Figures 2A–F**). Though MNK1 mRNA is marginally upregulated in breast cancer, MNK1 protein is significantly abundant in the cancer tissue (**Figure 2B**). Upregulation is evident at the protein level in all three genes, with breast tumor tissues from cancer patients exhibiting significantly higher levels of MNK1/2 and eIF4E (**Figures 2B, D, F**). Remarkably, elevated levels of eIF4E have been associated with poor overall survival in breast cancer patients (**Figure 2G**). Further, the analysis of patient data based on racial backgrounds indicated relatively higher levels of MNK1 protein in patients of African descent (**Figure 2H**). Among the major races represented in the database, Caucasian patients exhibited relatively higher levels of eIF4E expression in tumor tissues, followed by the African race (**Figure 2I**). These findings underscore the consistent dysregulation of MNK1/2 and eIF4E in breast cancer and provide insights into potential racial disparities in their expression patterns. It also emphasizes the importance of further investigations to unravel the underlying molecular mechanisms and implications in breast cancer disparities.

### 3.2 SYVN1 is constitutively upregulated in VNLG-152R-treated TNBC cells and associated with MNK1/2 degradation

**Figure 3:**
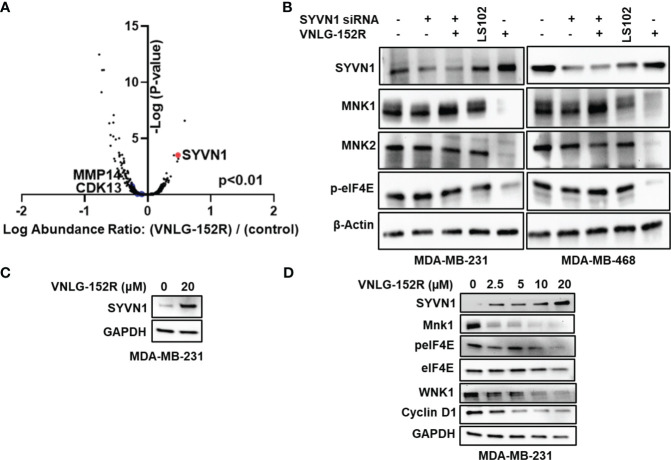
SYVN1 is upregulated in VNLG-152R-treated TNBC cells and correlated to MNK1/2 degradation. **(A)** Whole Proteome profiling by high-definition mass spectrometry (HD-MS) showed that SYVN1 is upregulated three-fold compared to the vehicle control (p<0.01). MDA-MB-231 cells were treated with 10 μM VNLG-152R for 24 h and processed as detailed in the methods section. **(B)** Degradation of MNK1/2 by VNLG-152R in QNBC is mediated by its ubiquitination by SYVN1. Immunoblots show upregulation of SYVN1 protein in VNLG-152-treated TNBC cells with concurrent degradation of MNK1/2. Knockdown of SYVN1 using siRNA or its inhibition by known inhibitor LS102 abrogated VNLG-152R-mediated degradation of MNK1/2 suggesting active role of SYVN1 in VNLG-152R-mediated MNK1/2 degradation in TNBC cells. As MNK1/2 are the only kinases known to phosphorylate eIF4E, a decrease in MNK1/2 levels further affects the level of p-eIF4E, thus limiting mRNA 5’cap-dependent translation initiation in TNBC cells. β-actin served as protein loading control. **(C)** Immunoblot showing upregulation of SYVN1 when MDA-MB-231 cells were treated with 20 μM VNLG-152R. **(D)** Dose-dependent effect of VNLG-152R on the expression of SYVN1, MNK1, eIF4E, peIF4E, WNK1 and Cyclin D1. MDA-MB-231 cells were treated with VNLG-152R (0-20 μM) for 24h. Cells were lysed with RIPA buffer and 40 μg of protein used in analyzing protein expression of, SYVN1, MNK1, eIF4E, peIF4E, WNK1 and Cyclin D1 respectively by immunoblotting. GAPDH was used as the loading control.

We first carried out the total proteome profiling of TNBC cells MDA-MB-231 using High-Definition Mass Spectrometry (HDMS) to visualize the differently expressed proteins upon treating with VNLG-152R (**Figure 1**). Among the differentially expressed proteins, SYVN1, an E3 ligase was found to be upregulated three-fold in the treated cells (**Figure 3A**). Based on our previous studies demonstrating the ubiquitin-proteasomal degradation of MNK1/2 induced by VNLG-152R in breast (9, 18) and prostate (33, 34) cancer cell lines and the role of SYVN1 as an E3 ligase involved in ubiquitination and proteasomal degradation of several proteins (35–42), we hypothesized that SYVN1 might play a key role in the ubiquitination and subsequent degradation of MNK1/2. Immunoblotting for SYVN1 confirmed its increased expression in VNLG-152R-treated cells compared to the control with concomitant decrease in MNK1/2 and its product p-eIF4E (**Figure 3B**). To further validate the involvement of SYVN1 in MNK1/2 degradation, we performed immunoblotting in MDA-MB-231 and MDA-MB-468 cells treated with VNLG-152R in the presence or absence of the SYVN1 inhibitor LS102 (43, 44) or SYVN1 siRNA. As predicted, VNLG-152R did not significantly affect the levels of MNK1/2 when SYVN1 inhibitor or siRNA was present, but it significantly decreased the levels of MNK1/2 in the absence of SYVN1 inhibitor or siRNA (**Figure 3B**). To address a concern raised by an astute reviewer, we note that LS102 is an inhibitor of the enzymatic activity of SYVN1 and hence we do not see (or expected) decreased levels of SYVN1. As MNK1/2 are the only known kinases known to phosphorylate eIF4E (13–15), the degradation of MNK1/2 by VNLG-152R was accompanied by decrease in eIF4E phosphorylation, indicating the active role of SYVN1 in the degradation of MNK1/2 mediated by VNLG-152R. Furthermore, we observed a dose-dependent increase in SYVN1 expression and a corresponding decrease in MNK1/2 and p-eIF4E levels upon treatment with increasing concentrations of VNLG-152R (**Figures 3C, D**). As expected, we also observed a dose-dependent decrease in other downstream oncoproteins involved in breast cancer cell migration, invasion, and cell cycle progression such as WNK1 (kinase with no lysine (K) 1) (45, 46), and Cyclin-D1 (47, 48), respectively (**Figure 3D**).

### 3.3 SYVN1 induces proteasomal degradation of MNK1/2 through ubiquitination

**Figure 4:**
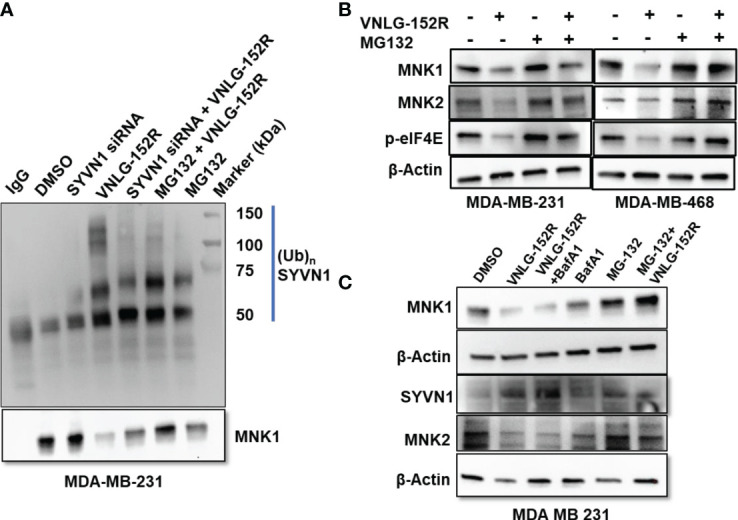
Inhibition of proteasomal degradation but not lysosomal degradation accumulates ubiquitinated MNK1. **(A)** MDA-MB-231 cells were treated with MG-132 prior to treating cells with VNLG-152R for short duration (2h). The cells were lysed and immunoprecipitated MNK1 using anti-MNK1 and probed with anti-ubiquitin. The cells treated with VNLG-152R resulted in accumulation of higher amount of ubiquitinated MNK1 compared to the controls. Short duration of treatment with VNLG-152R minimizes MNK1 degradation and facilitates maximum recovery of ubiquitinated MNK1. **(B)** Treatment of TNBC cells with proteasome inhibitor MG-132 in presence of VNLG-152R did not significantly alter the MNK1/2 levels, suggesting the proteasomal pathway of degradation of MNK1/2. Decrease in MNK1/2 is reflected by decreased levels of phosphorylated eIF4E. **(C)** MDA-MB-231 cells were treated with VNLG-152R in presence or absence of lysosome inhibitor Bafilomycin-A1 (BafA1) or proteasome inhibitor MG-132. Inhibition of lysosome by BafA1 did not abrogate VNLG-152R-mediated degradation of MNK1/2 but inhibition of proteasome by MG-132 abolished MNK1/2 degradation. This further suggests that the VNLG-152R-mediated degradation of MNK1/2 is through proteasomal pathway and not by lysosomal degradation. β-actin served as protein loading control.

After ascertaining the involvement of SYVN1 in the degradation of MNK1/2, we proceeded to investigate the ubiquitination of MNK1/2 through a proteasomal degradation inhibition assay. MDA-MB-231 cells were treated with MG-132, a known proteasomal inhibitor (49) prior to treating the cells with VNLG-152R briefly (2 h) to recover ubiquitinated MNK1/2, immunoblotted and probed with ubiquitin antibody. As expected, ubiquitinated MNK1/2 was accumulated in cells treated with MG-132 and VNLG-152R, while reduced ubiquitination was observed in cells treated with SYVN1 siRNA (**Figure 4A**). Thus, treating MDA-MB-231 and MDA-MB-468 cells with proteasome inhibitor MG132 in presence of VNLG-152R did not significantly alter the MNK1/2 levels, suggesting the proteasomal pathway of degradation of MNK1/2. The level of MNK1/2 was comparable to that of control when treated with MG-132 and VNLG-152R whereas we observed significant decrease of MNK1/2 in VNLG-152R treatment alone, further reenforcing the proteasomal degradation of MNK1/2 (**Figure 4B**).

However, there are two major pathways involved in degradation of cellular proteins *viz*. ubiquitin-proteasome system (UPS), which is specific in nature and associated with targeted protein degradation and more generic autophagy-lysosomal degradation that degrades protein aggregates and organelles, which is less specific but tightly regulated (50, 51). We ruled out the potential involvement of the autophagy-lysosomal pathway in the degradation of MNK1/2 by using Bafilomycin-A1 (Baf-A1), a standard inhibitor of lysosomal autophagy (52). Addition of Baf-A1 to cultured MDA-MB-231 cells did not inhibit VNLG-152R-mediated degradation of MNK1/2, indicating that the lysosomal pathway is not involved in the degradation of MNK1/2 mediated by VNLG-152R (**Figure 4C**). As expected, the levels of SYVN1 were elevated in the VNLG-152R treated cells (**Figure 4C**).

### 3.4 RNA-sequencing, GSEA and HD mass spectrometry-proteome profiling demonstrates inhibition of mTORC1 signaling and reveal pathways perturbed by VNLG-152R

**Figure 5:**
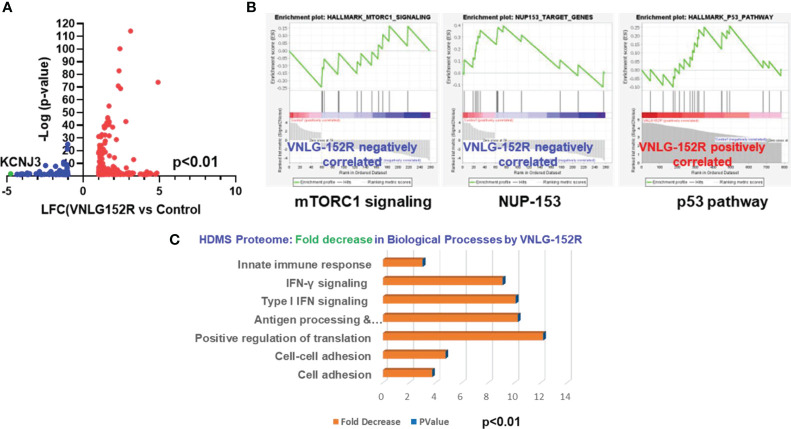
VNLG-152R modulates transcriptome and proteome of TNBC cells in favor of cancer inhibition. **(A)** RNA-sequencing and differential gene expression analysis show that 337 genes were differentially expressed when MDA-MB-231 cells were treated with 10 μM VNLG-152R for 24h; 259 genes were upregulated (red dots) and 78 downregulated (blue dots). **(B)** GSEA of differentially expressed genes demonstrate inhibition of MTORC1 and NUP-153 pathways but activation of p53 pathway by VNLG-152R. **(C)** Whole proteome profiling by high-definition mass spectrometry (HD-MS) of VNLG-152R-treated (10 μM for 24 h) MDA-MB-231 cells demonstrate modulation of several pathways. Notably, protein translation is inhibited 12-fold apparently due to MNK1/2 degradation besides inhibiting other biological processes such as cell adhesion to the matrix, cell to cell adhesion and IFN signaling critical to breast cancer progression. Statistical significance was computed at *P* < 0.01.

After establishing the role of SYVN1 in the degradation of MNK1/2 induced by VNLG-152R, we proceeded to assess the impact of VNLG-152R on canonical pathways relevant to breast cancer. To investigate the effect of 10 μM VNLG-152R on the cellular transcriptome of MDA-MB-231 cells, we conducted RNA sequencing and GSEA studies. Notably, VNLG-152R induced differential expression (DE) of 337 genes (**Figure 5A**). Gene Set Enrichment Analysis (GSEA) of these differentially expressed genes revealed the inhibition of key cancer pathways such as mTORC1 signaling and NUP153, while p53 was upregulated (**Figure 5B**). The inhibition of mTORC1 signaling is apparently due to MNK1/2 degradation by VNLG-152R, corroborating the biochemical data presented in the study. NUP153 (Nucleoporin 153) contributes to cell migration and proliferation and regulates the nuclear translocation of endothelial nitric oxide synthase (eNOS) by forming a multimeric complex (53). It is reported that eNOS is critical for maintaining tumorigenicity of cancer cells (54).

Further, the HDMS Proteome profiling and subsequent pathway analysis revealed a shift in total proteome of VNLG-152R-treated cells to reflect decreased levels of several pathway proteins involved in the biological processes such as cell adhesion to the matrix and cell-to-cell adhesion that are critical for breast cancer progression and cell migration were decreased 4-5-fold upon treating TNBC cells with VNLG-152R (**Figure 5C**). Particularly noteworthy, protein translation in the cells were decreased by 12-fold (**Figure 5C**), apparently due to the decreasing levels of MNK1/2 that is necessary for phosphorylating eIF4E, a pre-requisite for the formation of mRNA 5’ cap-dependent translation initiation complex eIF4F.

### 3.5 VNLG-152R and its deuterated analogs demonstrate potent inhibition of TNBC growth and inhibit tumor growth *in vivo* in diverse racial tumor xenograft models

#### 3.5.1 MDA-MB-231 tumor xenograft in mice: caucasian female patient with low/no AR

**Figure 6:**
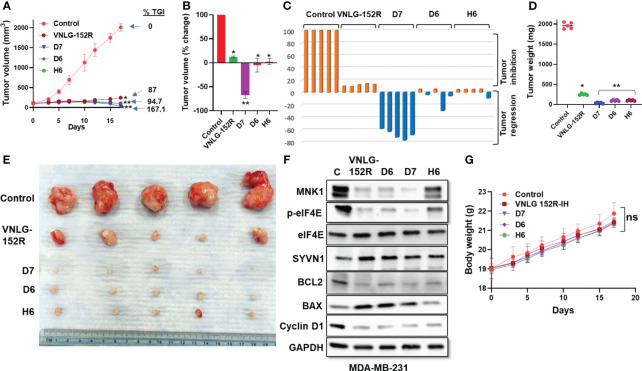
VNLG-152R and its deuterated analogs inhibit QNBC of Caucasian origin with no AR in NRG mice tumor xenograft model. MDA-MB-231 tumor xenografts were transplanted to NRG mice by subcutaneous injection of 5 x 10^6^ cells to the left flank. Oral administration of VNLG-152R and its deuterated analogs (20 mg/kg body weight, PO) significantly inhibited tumor growth and resulted in tumor regression without apparent host toxicity. **(A)** Tumor volume was measured periodically as indicated and plotted against time (days), all *p* values are compared to vehicle control: *P < 0.001, **P < 0.0001; **(B, C)** Comparison of percentage change in tumor volume of animals in the group and the individual mice in the group (waterfall plots); **(D)** All excised tumors were weighed and plotted for comparison of tumor mass. **(E)** Tumors excised from all the test animals at the end of the experiment and photographed; **(F)** Immunoblots of key proteins in the excised tumor tissue show significant reduction in MNK1/2 protein and decreased level of phosphorylated eIF4E. The level of SYVN1 is higher in the treated animals compared to the control. **(G)**. The body weights of mice periodically taken and plotted show no apparent host toxicity of the test compounds. *Statistics:* All *P* values are compared to vehicle control: *P < 0.001, **P < 0.0001.

Tumor xenografts in mice originated from the widely used TNBC model, MDA-MB-231 cell line, derived from pleural effusion of a 51-year-old Caucasian female with metastatic mammary adenocarcinoma is highly aggressive, metastatic, and fast-growing (55). Interestingly, many reports suggest that it lacks expression of AR protein though presence of AR mRNA is detected (22). When the mice bearing MDA-MB-231 tumor xenograft were orally administered with 20 mg/kg VNLG-152R, five days a week, it resulted in 87% tumor growth inhibition (TGI) as measured by tumor volume (**Figures 6A–C**). Remarkably, the deuterated analogs D6, D7, and H6 demonstrated even higher tumor growth inhibition (94% each for D6 and H6, respectively), with D7 causing *67% tumor regression* compared to the initial tumor volume. The percentage change in tumor volume was plotted for the groups (**Figure 6B**) and for the individual animals in the groups (**Figure 6C**) show significant tumor regression in D7-treated group and 1-3 animals in the D6- and H6-treated groups. The weight of excised tumors was plotted and corresponded to the tumor volume (**Figure 6D**). **Figure 6E** shows the photograph of all the excised tumors after termination of the study which corroborates the tumor volumes shown in **Figures 6A-C**. Immunoblotting analysis of excised tumor tissue revealed significantly decreased levels of MNK1, accompanied by increased expression of SYVN1, confirming the expected molecular response to treatment (**Figure 6F**). Moreover, downregulation of the antiapoptotic protein BCL2, upregulation of the pro-apoptotic protein BAX, and decreased expression of Cyclin D1, crucial for cell cycle progression, were observed in the treated tumor tissues of the Caucasian model of TNBC/QNBC *in vivo*. We did not assess the levels of AR in the MDA-MD-231 tumors because we (*data not shown*) and others have shown that MDA-MB-231 cells have undetectable level on AR protein (22). Importantly, the body weight of the control and treated animals did not show significant differences, indicating the absence of treatment-induced toxicity of the test molecules at the given dose (**Figure 6G**).

#### 3.5.2 MDA-MB-468 tumor xenograft in mice: female patient of African descent with low/no AR

**Figure 7:**
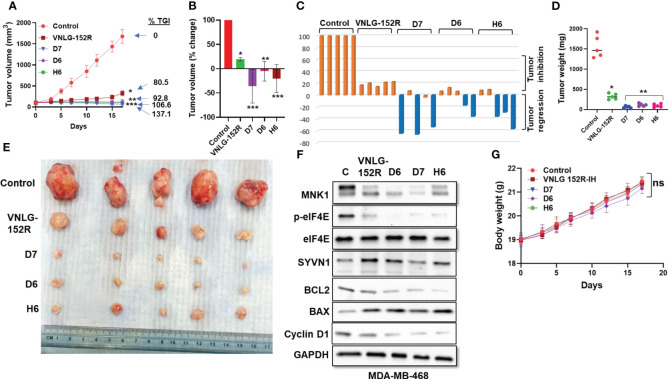
VNLG-152R and its deuterated analogs effectively inhibit TNBC of African origin with low or no AR expression *in vivo* in NRG mice. NRG mice were subcutaneously injected with 5x10^6^ MDA-MB-468 cells in the left flank to establish tumor xenografts. Oral administration of VNLG-152R and its deuterated analogs (20 mg/kg body weight, PO) effectively suppressed tumor growth and induced tumor regression. **(A)** Tumor volume was periodically measured and plotted over time to assess the growth of tumors in response to the treatments; **(B, C)** Percentage change in volume of tumor from all animals in the group and that of individual mice (waterfall plots). **(D)** All excised tumors were weighed and plotted for comparison of tumor mass. **(E)** Tumors excised from all the test animals at the end of the experiment and photographed; **(F)** Immunoblots of proteins of interest in the excised tumor show reduction in level of MNK1/2. Further, the level of phosphorylated eIF4E is decreased and SYVN1 is higher in the tissue of treated animals. **(G)** The body weights of mice periodically taken and plotted show no signs of host toxicity of the test compounds. *Statistics:* All *P* values are compared to vehicle control: *P < 0.01, **P < 0.001, **P < 0.0001.

MDA-MB-468 cell line is derived from metastatic adenocarcinoma of the breast from a female patient of African ancestry, expresses no AR and characterized by aggressive lymphatic metastasis (56). In mice tumor xenograft model of MDA-MB-468, oral administration of 20 mg/kg VNLG-152R and its deuterated analogs (D6, D7, and H6) resulted in significant inhibition of tumor growth. VNLG-152R inhibited tumor growth by 80.5%, while D6 exhibited a higher inhibition rate of 92.8%. Remarkably, both D7 and H6 completely inhibited tumor growth and induced *tumor regression* by 37.1% and 6.6%, respectively (**Figures 7A–C**). Importantly, the percentage change in tumor volume demonstrated significant tumor regression in at least two mice in the groups treated with the deuterated analogs (**Figures 7A-C**). The weights of the excised tumors from all animals were consistent with the tumor volume, further confirming the reduction in tumor mass following treatment with VNLG-152R or its analogs (**Figure 7D**). Furthermore, the oncogenic proteins BCL2 and Cyclin D1 were downregulated, while the proapoptotic protein BAX was upregulated in the excised tumors treated with VNLG-152R and the deuterated analogs (**Figure 7F**). Immunoblotting analysis of key proteins in the excised tumor tissue revealed significant downregulation of MNK1, accompanied by a decrease in p-eIF4E, which can be attributed to elevated levels of SYVN1 compared to the control (**Figure 7C**). Furthermore, the oncogenic proteins BCL2 and Cyclin D1 were downregulated, while the pro-apoptotic protein BAX was upregulated in the excised tumors treated with VNLG-152R and the deuterated analogs. As with the MDA-MB-231 tumors, we did not assess the impact of treatments on AR as the MDA-MB-468 cells do not express detectable levels of AR (22). Throughout the study period, the body weight of the animals did not show any significant changes in the treatment groups compared to the control, indicating that the administered compounds were not associated with significant toxicity at the given dose (**Figure 7G**).

#### 3.5.3 MDA-MB-453 tumor xenograft in mice: caucasian female patient with high AR

**Figure 8:**
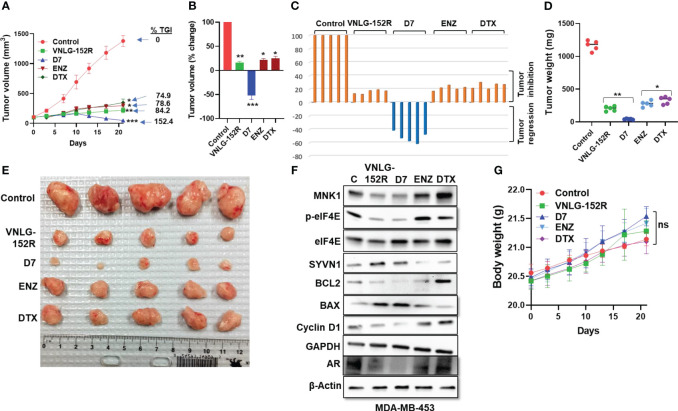
VNLG-152R and its deuterated analogs inhibit TNBC of Caucasian origin with high AR in NRG mice tumor xenograft model. MDA-MB-453 tumor xenografts were transplanted to NRG mice by subcutaneous injection of 3x10^6^ cells to the left flank. Oral administration of VNLG-152R and its deuterated analog D7 (20 mg/kg body weight, PO) significantly inhibited tumor growth and resulted in tumor regression without apparent host toxicity. DTX was administered by IP injection (5 mg/kg body weight). **(A)** Tumor volume was measured periodically as indicated and plotted against time (days) and shows significant inhibition of tumor growth in treated animals. Notably, D7 caused 52.4% tumor regression. **(B, C)** Percentage change in tumor volume of mice in different groups and that of the individual animals (waterfall plots). **(D)** All excised tumors were weighed and plotted for comparison of the tumor mass. **(E)** Tumors excised from all the test animals at the end of the experiment and photographed. **(F)** Immunoblots of putative proteins in the excised tumor tissue show a decrease in MNK1/2 and consecutive reduction of p-eIF4E level in the tumor of treated animals. **(G)** The body weights of mice periodically taken and plotted show no apparent host toxicity of the test compounds as there is no significant difference in body weights. *Statistics:* All *P* values are compared to vehicle control: *P < 0.01, **P < 0.001, **P < 0.0001.

Finally, we tested the antitumor efficacy of VNLG-152R and its most potent deuterated analog, D7 in MDA-MB-453 xenograft tumor model in female NRG mice in head-to-head comparison with Enzalutamide (ENZ) and Docetaxel (DTX). It is noteworthy that unlike a recent report which found that MDA-MB-453 tumors grew very slowly in either female or male SCID mice (22), our study clearly established that MDA-MB-453 xenograft tumors grew exceptionally well in female NRG mice (**Figure 8A**). MDA-MB-453 cell line represents a type of aggressive TNBC and was originally developed from metastatic breast cancer of a Caucasian female patient with metastatic sites involving the nodes, brain and both pleural and pericardial cavities (57). Unlike the other TNBC models investigated in this study, MDA-MB-453 expresses high levels of AR (22). Despite being less proliferative in nature, the LAR (luminal androgen receptor) subtype of TNBC is less responsive to chemotherapy than the basal type (58–60). When the mice transplanted with MDA-MB-453 tumor xenografts were treated with VNLG-152R and its deuterated analog D7, tumor growth was significantly inhibited as shown by the tumor volume (**Figures 8A-C**). VNLG-152R exhibited 84.2% inhibition of tumor growth, while D7, the most promising analog in other models, completely inhibited tumor growth and led to a remarkable 52.4% *tumor regression*. As anti-androgen therapy is a preferred clinical treatment option in AR-positive TNBC (7, 61–64), we compared the test compounds with clinically relevant anti-androgen, ENZ and chemotherapeutic, DTX, which inhibited tumor growth by 78.6% and 74.9%, respectively (**Figures 8A–C**). *It is important to state here that ENZ* (65) *and DTX* (66) *were administered at their optimal preclinical dosing regimens, and, it should be noted that higher doses of DTX have been shown to be toxic to mice.* The percentage change in tumor volume for individual animals and the treatment group is presented in **Figures 8B, C**, highlighting significant *tumor regressions* in all animals of the D7-treated group with a mean value of 52.4%. The weights of the excised tumors from all animals corresponded to the tumor volumes, providing further confirmation of the decrease in tumor mass after treatment with VNLG-152R or its analog (**Figure 8D**). **Figure 8E** shows the photograph of all the excised tumors after termination of the study which corroborates the tumor volumes shown in **Figures 6A–C**. Immunoblotting analysis of key proteins in the excised tumor tissue revealed upregulation of SYVN1 and a concomitant decrease in MNK1, resulting in reduced levels of p-eIF4E, modulation of apoptosis (BAX/BCL-2 ratios), depletion of cyclin D1 like the observations in other TNBC models (**Figure 8F**). In this model, and as expected (33, 34, 67), we also observe significant depletion of AR in tumors treated with VNLG-152R and D7 (**Figure 8F**). Consistent with the other studies, the tested compounds did not exhibit any toxic effects on the animals at the studied dose, as evidenced by stable body weight throughout the study period (**Figure 8G**).

## 4 Discussion

The pharmacological intervention of TNBC is an intricate challenge due to its diverse sub-types and unique molecular signatures, each presenting its own complexities. Additionally, patients of different racial backgrounds respond differently to available drugs. the pharmacological outcome is largely dependent on molecular signatures and ethnicity, with the African women registering the least overall survival (68). Due to this racial disparity in overall survival and response to drugs, it is imperative to study the efficacy of novel putative drugs in *in vivo* models of TNBC representing different racial origin.

The standard treatment regimens with hormone or HER2-targeted therapies are not an option in treating TNBC/QNBC patients (69). One of the alternative strategies is to pharmacologically target dysregulated translation machinery in the tumor cell as demonstrated by inhibition of MNK1/2 by eFT508 and other agents (70, 71). Notably, MNK1/2 are the only kinases known to phosphorylate eIF4E critical for the formation of translation initiation complex eIF4F (13–15). Since MNK1/2 are at the crossroads of other signaling pathways vital for the cancer development and progression such as mTORC1-4E-BP1 signaling and eIF4E signaling axes (16, 72), restraining MNK1/2 significantly inhibits cancer cells proliferation, cell migration, invasion, and metastasis (71).

**Figure 9:**
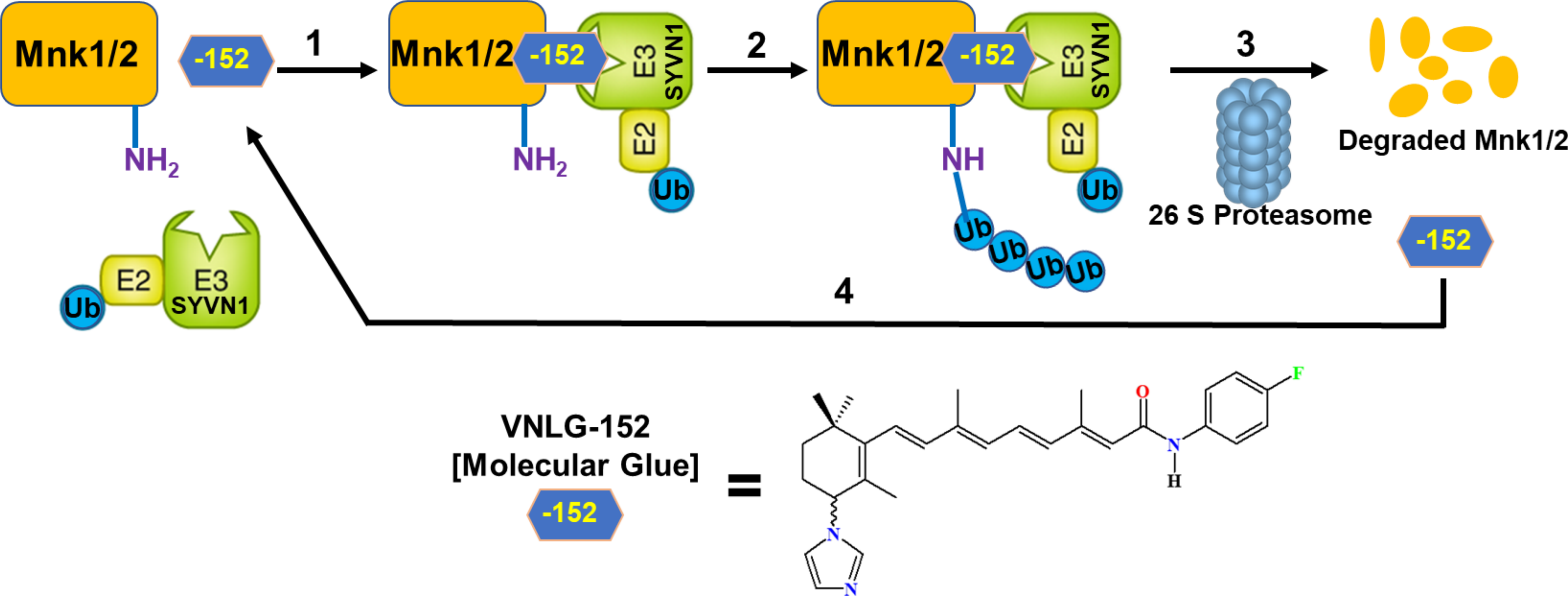
Schematic representation of mechanism of action of VNLG-152R in inhibiting TNBC by degrading MNK1/2. A ternary complex formed upon binding of VNLG-152R (-152; Molecular Glue), target proteins (MNK1/2) and SYVN1 E3 ligase complex (step 1), to promote MNK1/2 protein ubiquitination (Ub) (step 2), followed by MNK1/2 degradation by the proteasome and release of VNLG-152R (step 3). Free VNLG-152R can then bind another molecule of MNK1/2 (step 4) to repeat the degradation cycle.

The present study further extends our current understanding of the benefits of pharmacologically targeting MNK1/2 in TNBC/QNBC and unravels the molecular mechanism of VNLG-152R-mediated degradation of MNK1/2. Transcriptome and proteome-guided study further suggested the role of E3 ligase SYVN1 in MNK1/2 degradation. Interestingly, biochemical, and molecular studies emphasized the involvement of SYVN1 in ubiquitination of MNK1/2 as the presence of SYVN1 inhibitor LS102 or siRNA-knockdown of SYVN1 abolished the MNK1/2 degradation by VNLG-152R. Furthermore, immunoblots showed the presence of elevated levels of ubiquitinated MNK1/2 upon VNLG-152R treatment when proteasome was inhibited using the known proteasome inhibitor MG-132, suggesting the proteasomal degradation of ubiquitinated MNK1/2. VNLG-152R and the analogs might act as a molecular glue that brings together SYVN1 and MNK1/2 facilitating proximity-induced ubiquitination and subsequent proteasomal degradation as depicted in **Figure 9**. This study, including our previous studies (9, 18, 19, 33, 34), clearly establishes VNLG-152R and its analogs as monomeric molecular glues that induce MNK1 and MNK2 ubiquitin-proteasomal degradation to inhibit oncogenic eIF4F complex.

It is well established that E3 ligases, including SYVN1 can have opposite effects as either tumor suppressors (TS) or oncogenes depending on the context or type of cancer (73–75). With regards to SYVN1, previous studies have shown that it functions as a tumor suppressor in breast (37, 39, 41, 76, 77) and ovarian (40) cancers. On the contrary, the tumor-promoting (oncogenic) effects of SYVN1 have been revealed in colon cancer (78), lung cancer (79, 80), and hepatocellular carcinoma (81, 82).

Another significant finding of this study is that VNLG-152R caused dose-dependent depletion of WNK1 (**Figure 3D**) which is implicated in cell migration, invasion, and metastasis in multiple cancer types including glioblastoma (83), prostate cancer (84), non-small cell lung cancer (85), and breast cancer (45, 46, 86, 87). Because metastasis is the major cause of mortality in patients with breast cancer (88), we posit that our compounds can be developed as small molecules therapeutics with the characteristics of inhibiting both cell proliferation and metastasis, which would undoubtedly have a major impact on mortality in patients with breast cancer.

Transcriptome and proteome analyses further demonstrate inhibition of oncogenic pathways such as mTORC1 and NUP152 signaling in TNBC cells treated with VNLG-152R. Our study reveals a remarkable finding that VNLG-152R and the deuterated analogs are capable of inhibiting TNBC in patients of different ethnicity and molecular signatures. This includes patients with higher levels of MNK1 and eIF4E expression in tumors irrespective of AR expression status. Besides delineating the molecular mechanism of action of VNLG-152-induced degradation of MNK1 and MNK2, we clearly demonstrate that VNLG-152R and its deuterated analogs effectively inhibit TNBC tumor xenografts of both Caucasian and African origins, including those with low or no AR expression as well as the Caucasian race with high AR expression.

## 5 Conclusion

In conclusion, we have clearly established that SYVN1 is the prime E3 ligase implicated in the VNLG-152/deuterated analogs-induced ubiquitin-proteasomal degradation of MNK1 and MNK2 degradation *in vitro* and *in vivo*. Because SYVN1 has been shown to act as a tumor suppressor in TNBC models, *in vitro* and *in vivo*, we propose that this phenomenon may also contribute to the anti-tumor efficacy of our compounds. Indeed, the inhibition of MNK1/2-mediated eIF4E phosphorylation reduces the formation of the translation initiation complex eIF4F, effectively restraining dysregulated protein synthesis central to tumor growth, progression and metastasis. Our findings highlight the significant potential of VNLG-152R and its deuterated analogs in effectively combating TNBC/QNBC across patients of diverse racial backgrounds, regardless of their genetic background and AR expression status. These results emphasize the broad applicability and efficacy of these compounds in addressing the challenges associated with TNBC/QNBC treatment in a racially diverse population. The data presented here clearly justify the on-going Investigational New Drug (IND) studies with VNLG-152R under the auspices of Isoprene Pharmaceuticals, Inc., in view Phase I clinical trials in women with TNBC and solid tumors.

## Data availability statement

The original contributions presented in the study are publicly available. This data can be found here: GEO repository, accession number GSE242514.

## Ethics statement

Ethical approval was not required for the studies on humans in accordance with the local legislation and institutional requirements because only commercially available established cell lines were used. The animal study was approved by Institutional Animal Care and Use Committee (IACUC), University of Maryland School of Medicine, Baltimore, MD, USA, per IACUC No. # 0221010 dated 03/09/2021. The study was conducted in accordance with the local legislation and institutional requirements.

## Author contributions

The study was conceptualized and designed by RT, ET and VN. RT, ET and PP performed the experiments and acquired data. The data was analyzed by RT, ET, DW, VR, WH, MK and VN. RT, ET and VN wrote the manuscript. VN supervised the entire study. All authors contributed to the article and approved the submitted version.
